# Research priorities to support typhoid conjugate vaccine decision-making in India: evidence assessment and stakeholder survey

**DOI:** 10.1136/bmjph-2024-001089

**Published:** 2024-10-03

**Authors:** Vijayalaxmi V Mogasale, Anish Sinha, Jacob John, Habib Hasan Farooqui, Arindam Ray, Tracey Chantler, Vittal Mogasale, Bhim Gopal Dhoubhadel, W John Edmunds, Andrew Clark, Kaja Abbas

**Affiliations:** 1 Department of Infectious Disease Epidemiology and Dynamics, London School of Hygiene & Tropical Medicine, London, UK; 2 School of Tropical Medicine and Global Health, Nagasaki University, Nagasaki, Japan; 3 Department of Public Health Science, Indian Institute of Public Health Gandhinagar, Gandhinagar, Gujarat, India; 4 Department of Community Health & Development, Christian Medical College Vellore, Vellore, Tamil Nadu, India; 5 College of Medicine, Qatar University, Doha, Qatar; 6 Department of Infectious Disease & Vaccine Delivery, Bill & Melinda Gates Foundation India, New Delhi, India; 7 Department of Global Health and Development, London School of Hygiene & Tropical Medicine, London, UK; 8 Graduate School of Public Health, Yonsei University, Seoul, Korea (the Republic of); 9 Institute of Tropical Medicine, Nagasaki University, Nagasaki, Japan; 10 Department of Health Services Research and Policy, London School of Hygiene & Tropical Medicine, London, UK; 11 Public Health Foundation of India, New Delhi, India

**Keywords:** Public Health, Epidemiology, Disease Transmission, Infectious, Vaccination, Public Health Practice

## Abstract

**Background:**

India has a high typhoid fever burden. In 2022, the National Technical Advisory Group on Immunisation recommended introducing typhoid conjugate vaccine (TCV) into the Universal Immunisation Programme. Our study aims to identify research priorities to support ongoing TCV decision-making in India.

**Methods:**

We identified 45 evidence factors for TCV decision-making in India by adapting WHO’s Evidence-to-Recommendation framework. We assigned an evidence gap score for each evidence factor from 0 (low) to 4 (high) based on the availability and sufficiency, quality, breadth and applicability of evidence identified in a literature review (end date 30 November 2023). We assigned each evidence factor an importance score based on the results of an online survey conducted among national immunisation stakeholders (n=22, 1 July 2023–31 October 2023), where they ranked the importance of seven WHO’s Evidence-to-Recommendation criteria and several evidence factors within them. We rescaled mean stakeholder rankings into importance scores from 0 (low) to 4 (high). Finally, we added the evidence gap score to the importance score and used the overall scores to identify research priorities to support ongoing TCV decision-making in India.

**Results:**

We estimated the highest evidence priority scores for public perception of typhoid fever, vaccination budget impact, vaccine availability, socioeconomic impact, fiscal space, antimicrobial resistance tracking, typhoid fever mortality, public perception of TCV, immunisation managers’ acceptance and vaccine schedule preferences among caregivers.

**Conclusion:**

By adapting WHO’s Evidence-to-Recommendation framework to the Indian context, we systematically identified several research priorities to support ongoing decision-making on TCV in India. These priorities will evolve as new research studies and questions emerge about the optimal scheduling, roll-out and implementation of TCV in India.

**WHAT IS ALREADY KNOWN ON THIS TOPIC**

- India has a high typhoid fever burden in children, particularly in urban areas.
- Four licensed typhoid conjugate vaccines (TCVs) known to be safe and effective are available in the private sector in India and can be used in typhoid fever control.
- India’s National Technical Advisory Group on Immunisation has recommended using TCV in the Universal Immunisation Programme in 2022; an evidence-informed strategy and implementation plan have yet to be developed.

**WHAT THIS STUDY ADDS**

- The evidence gaps identified through the literature assessment are the target population’s vaccination schedule preferences, vaccine hesitancy and fiscal space analysis.
- The stakeholders perceived typhoid fever incidence, vaccine efficacy, severity, antimicrobial resistance and vaccine safety as the most important evidence.
- The research priorities (evidence gaps with high stakeholder importance) were identified in three domains: acceptability (to health staff and the public), financial considerations (budget impact, fiscal space) and the health and economic burden of typhoid fever (socioeconomic burden, typhoid mortality and antimicrobial resistance).

**HOW THIS STUDY MIGHT AFFECT RESEARCH, PRACTICE OR POLICY**

- We identified India-specific research priorities to support TCV decision-making in India by combining the metrics for evidence gaps and stakeholders’ perceived importance. This will give impetus to the key research essential for TCV decision-making in India.
- The novel method developed in our study to adapt the WHO’s Evidence-to-Recommendation framework to the country context to generate a decision-stage appropriate evidence-priority league table to support decision-making can be applied to new vaccine introduction and implementation in India and different settings.

## Introduction

 Typhoid fever is a multisystem febrile illness caused by *Salmonella enterica* serovar Typhi.^1^ The estimated global incidence of typhoid fever ranges from 11 to 21 million cases and 130 000 to 220 000 deaths annually in studies published from 2014 to 2019.^2 3^ India is a typhoid fever high-burden country, and the population-based incidence estimates range from 35 to 1173 per 100 000 person-years from 2017 to 2020.^4 5^ A geospatial model has estimated a national incidence of 360 cases (95% CI 297 to 494) per 100 000 person-years, with state-wise incidence ranging from 149 to 1245 cases per 100 000 person-years and a total of 4.5 (95% CI 3.7–6.1) million cases and 8930 (95% CI 7360–12 260) deaths annually with a case fatality ratio of 0.2% from 2017 to 2020.^6^ With effective antimicrobial use, the case fatality ratio of typhoid fever is <1%,^1^ but the rising antimicrobial resistance worldwide and reporting of extensive drug resistance have caused global concerns and given impetus to using the typhoid conjugate vaccine (TCV) for typhoid fever prevention and control.^7^

The WHO issued a vaccine position paper in March 2018 recommending evidence-informed use of TCV in routine immunisation programmes in priority settings based on TCVs’ improved immunogenic properties, suitability in younger children and expected longer duration of protection among available typhoid vaccines.^8^ Also in 2018, Gavi, the Vaccine Alliance has committed to supporting the use of TCV in eligible countries and has earmarked US$85 million for this purpose,^9^ followed by continued support for using TCV as part of their vaccine portfolio.^10^ From 2019 to 2023, six countries, namely Pakistan, Liberia, Zimbabwe, Samoa, Nepal and Fiji, have implemented TCV nationally in their routine immunisation programmes for children at 9 months or 15–18 months.^11^

Four licensed TCVs, of which two are WHO prequalified, are available in the Indian private market.^12^ In 2022, India’s National Technical Advisory Group on Immunisation (NTAGI) recommended introducing TCVs in the Universal Immunisation Programme (UIP) (national immunisation programme of India).^13^ A plan for the phased introduction of TCV needs to be developed based on various decision criteria and operational considerations.^13^ As seen for other new vaccine introductions in India, national-scale implementation decision-making of TCV may take several years with the continued generation of specific evidence, preparedness and phased roll-out. For example, NTAGI recommended the rotavirus vaccine in 2014, but its phased implementation in India started in 2016^14^ and was completed by 2020.^15^

The evidence requirements for new vaccine decision-making are complex, dynamic and resource-intensive. Continued evidence generation is needed in various decision-making phases for the roll-out of a vaccine, whether developing recommendations for introduction or planning implementation. Therefore, identifying research priorities using a systematic approach to address evidence needs is crucial for the optimal utilisation of research resources.

For a systematic approach, the WHO has listed essential criteria and linked factors for national immunisation technical advisory groups to consider for decision-making on the introduction of new vaccines.^16^ There are 7 criteria linked with 26 evidence factors in this ‘Evidence-to-Recommendation (EtR) framework’.^17 18^ These EtR criteria include disease burden (problem), benefits and harms of the intervention, values and preferences of the target population, acceptability to stakeholders, resource use, equity and feasibility. By adapting the WHO-EtR framework to the Indian context, our study aims to identify research priorities for ongoing decision-making on TCV in India by systematically assessing the evidence gaps and stakeholders’ perceived importance.

## Methods

**Figure 1:**
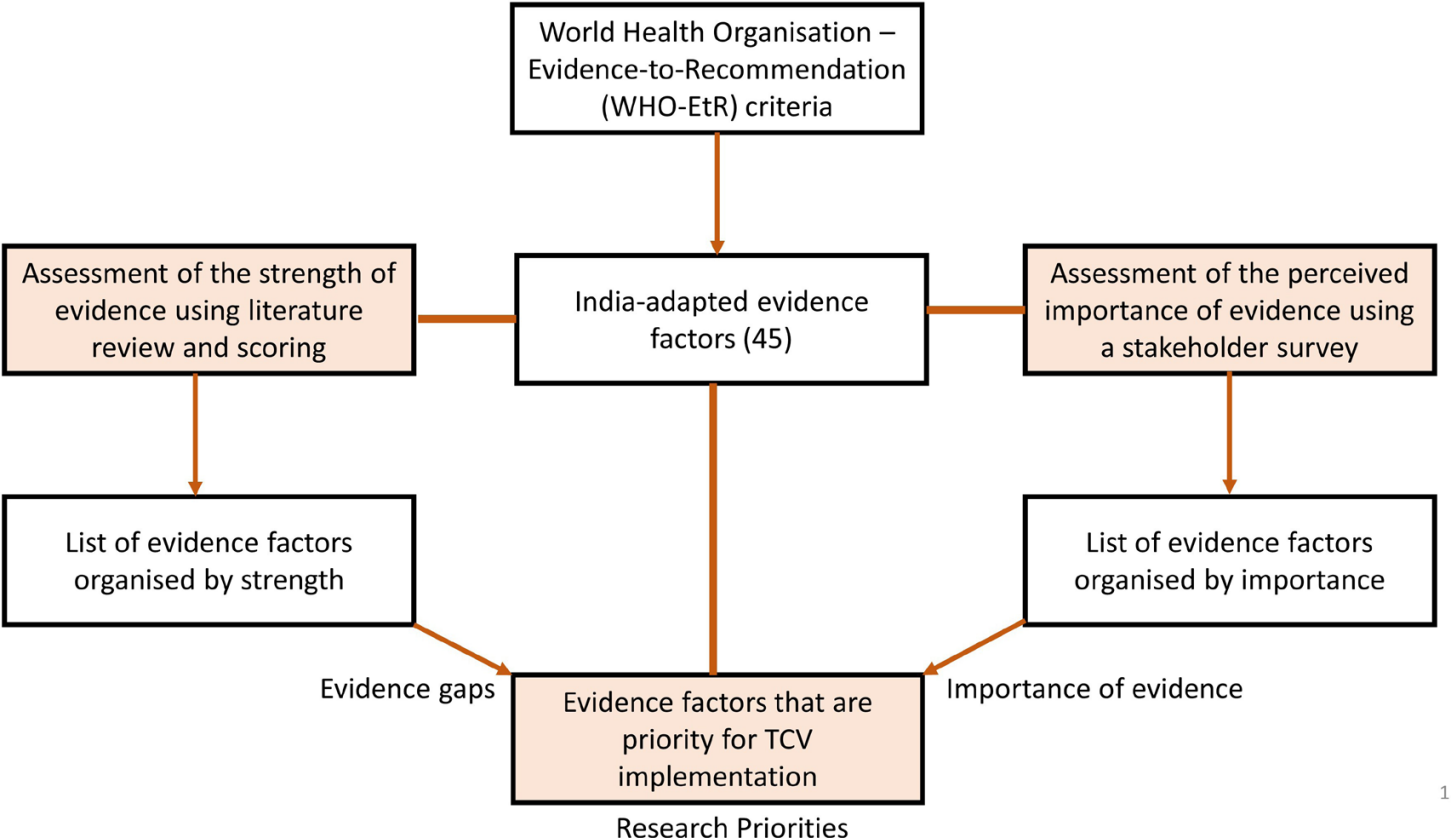
Evidence prioritisation**.** India-adapted evidence factors were developed using WHO-EtR framework, and research priorities to support decision-making on typhoid conjugate vaccine (TCV) in India were identified by assessing the evidence gaps and stakeholders’ perceived importance.

Based on a literature review, we adapted the WHO-EtR framework and streamlined evidence criteria and factors for supporting TCV decision-making in the UIP of India. We scored the strength of existing evidence in the literature for each criterion and related factors to assess evidence gaps. Based on a survey of key stakeholders, we assessed the perceived importance of the same criterion and related evidence factors. Finally, we combined the evidence gaps with key stakeholders’ perceived importance to identify research priorities for generating new evidence to support decision-making for TCV in India (figure 1).

### Literature review and adaption of WHO-EtR framework to India

We conducted a literature review to identify Indian data relevant to the criteria listed under the WHO-EtR framework.^17 18^ The search included peer-reviewed publications from the PubMed database (search 1: “typhoid*” and “India”; search 2: “typhoid conjugate vaccine”) and grey literature from the clinical trials registries, WHO-SAGE (Strategic Advisory Group of Experts on Immunisation) background documents, India-specific NTAGI meeting minutes and Ministry of Health website and personal contact with researchers working on typhoid fever in India until 30 November 2023 (online supplemental annex 1a). More details about the literature review can be found here.^19^ We expanded 26 WHO-EtR evidence factors into India-specific ones based on the literature review and added other factors from the meeting minutes of the NTAGI^13^ for a total of 45 evidence factors (online supplemental annex 1b).

### Assessment of the strength of existing evidence to identify evidence gaps

We assessed the strength of existing evidence derived from the summarised literature review under India-adapted evidence factors based on three evidence attributes adapted from ‘Systems to Rate the Strength of Scientific Evidence’.^20 21^ These three attributes were availability and sufficiency of evidence (eg, number of studies and sites), quality of evidence (eg, study design, inconsistencies, biases), and breadth and applicability of evidence (eg, age/state/urban–rural stratification) represented by three questions described in detail in online supplemental annex 2. We adapted the GRADE scoring approach^22 23^ with 0–4 for each of the three attributes, with 1–4 mapping to GRADE levels of certainty, and added option ‘zero’ (0) to indicate where evidence is unavailable (online supplemental annex 2). Thereafter, we calculated the mean score for three questions for each evidence factor to estimate the respective strength score (SS) in 5 levels ranging from 0 to 4 scores. We converted the SS into an evidence gap score (GS) ranging from 4 to 0, where 4 is the highest evidence gap and 0 is no evidence gap (GS=4–SS, see box 1).

Box 1 **Calculation process to identify evidence (research) priorities**

**Step 1. Evidence gap score (GS)**

 GS_k_=4−SS_k_

 SS_k_ refers to the evidence strength score for the k^th^ evidence factor, assessed from the literature on a scale of 0–4.

**Step 2. Evidence factor score (ES)**

 ES_k_=RS_k_×RW_j_

 RS_k_ refers to the mean rank score for the k^th^ evidence factor.

 RW_j_ refers to the mean rank (weight) for the j^th^ EtR criteria (corresponding to the k^th^ evidence factor).

**Step 3. Evidence importance score (IS)**

 IS_k_=4×((max(ES)−ES_k_)/(max(ES)−min(ES)))

 IS_k_ refers to the inverse normalised importance score for the k^th^ evidence factor.

 ES_k_ refers to the evidence factor score of k^th^ evidence factor.

 max(ES) refers to the maximum evidence factor score.

 min(ES) refers to the minimum evidence factor score.

**Step 4. Evidence priority score (PS)**

 PS_k_=GS_k_+IS_k_

 GS_k_ refers to the evidence gap score for the k^th^ evidence factor.

 IS_k_ refers to the inverse normalised importance score for the k^th^ evidence factor.

### Stakeholder survey to assess perceived importance of evidence

#### Stakeholders’ selection

**Figure 2:**
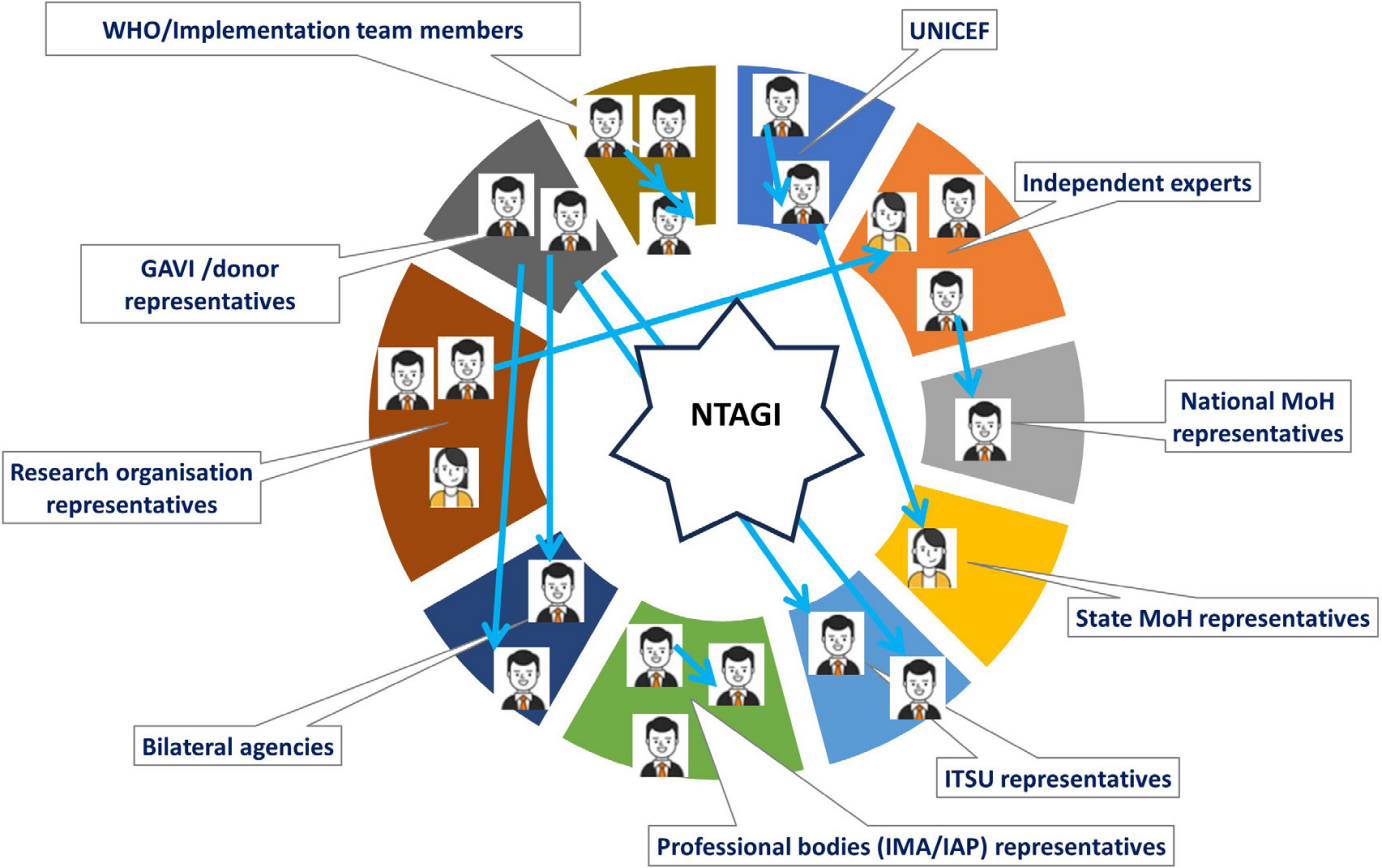
Stakeholders. Key stakeholders were identified using purposive and snowball sampling to participate in our survey. The stakeholders were organised into 10 groups for a total of 22 stakeholders of both genders (3 women and 19 men). The survey started with researchers’ known contacts who represented initial stakeholders, and the blue arrow indicates subsequent referrals by stakeholders. IAP, Indian Academy of Paediatrics; IMA, Indian Medical Association; MoH, Ministry of Health; NTAGI, National Technical Advisory Group on Immunisation.

We contacted 46 stakeholders from India, and 22 of them participated in our survey (online supplemental annex 3), using purposive and snowball sampling^24^ (online supplemental annex 4). These were the key stakeholders involved in vaccine introduction decisions through direct or indirect interaction with NTAGI on various occasions and were representative of key stakeholder groups—National Ministry of Health, State Ministry of Health, Immunisation Technical Support Unit, Indian Academy of Paediatrics, Gavi, UNICEF, WHO, bilateral agencies, research organisations and independent experts (see figure 2, online supplemental annex 3). The stakeholders’ expertise ranged from health policy, public health, epidemiology, vaccine delivery, health economics and social sciences to ethics (online supplemental annex 3). We organised the potential stakeholders into ten groups (figure 2) based on NTAGI meeting minutes^13^ and represented participants from each group.

#### Survey tool

We represented WHO-EtR criteria and India-adapted evidence factors in question format in the survey tool developed in Microsoft Forms online (see online supplemental annex 5). We requested key stakeholders to rank-order WHO-EtR criteria and various India-adapted evidence factors from their perspectives, irrespective of the availability of evidence. The first question comprised seven WHO-EtR criteria (as seven subquestions), and the following questions contained India-adapted evidence factors under each EtR criteria. The survey also included three open-ended policy questions matching NTAGI recommended vaccination strategies for TCV implementation in India (1) optimal age scheduling of TCV, (2) state-wise roll-out of TCV and (3) additional school-based TCV.

We pilot tested the tool with five Indian stakeholders and refined it based on their feedback before implementation. We conducted the survey between 1 July 2023 and 31 October 2023 (see online supplemental annex 3 for sampling details and the survey process).

#### Data analysis

We estimated the mean rank from stakeholder rankings to assess their prioritisation for each of the seven WHO-EtR criteria. The mean rank ranges from 1 to 7, and we used it as a rank weight (RW) specific to the WHO-EtR criteria as it encompassed all evidence factors within that criterion. We then estimated the mean rank from stakeholder ranking for each evidence factor organised under each of the seven WHO-EtR criteria. The rank order of each evidence factor was used as a rank score (RS) to estimate the stakeholders’ prioritisation for that evidence factor (under each of the seven WHO-EtR criteria). Thus, each evidence factor had an RW derived from the ranking of WHO-EtR criteria and an RS derived from its ranking within the WHO-EtR criteria. We multiplied the RS of India-adapted evidence factors with respective RW to obtain an evidence factor score (ES) (see) following the principle of multiple criteria decision analysis (MCDA).^25 26^ As this ES was a multiplier of rank orders, the lower the score indicates the higher the importance of the evidence factor. We inverse-normalised the ES between 0 and 1 and then multiplied it by 4 to convert it to a scale between 0 and 4 to estimate the importance score (IS) (matching the scale of evidence gap score GS). Higher IS indicate higher perceived importance by the stakeholders.

We organised the responses to open-ended questions by themes under each question, and total responses were counted for each theme under respective questions to identify the most important factors.

### Defining evidence priorities

We summed the evidence GS (scale of 0–4) and perceived IS (scale of 0–4) to estimate an evidence (research) priority score (PS=GS+IS), scale of 0–8 and colour-coded them after classifying by quintiles. Higher evidence PS indicate higher research priority to generate this evidence.

## Results

### Assessment of the strength of existing evidence to identify evidence gaps

We inferred robust evidence for alternative typhoid control measures; TCV efficacy, safety and effectiveness; acceptability to WHO, donors, NTAGI and professional bodies; value for money; and implementation feasibility from the perspective of human resources, storage capacity, adverse event following immunisation monitoring, health management information system, historical vaccination coverage (table 1). We found high evidence gaps (GS=4.00/4.00) for vaccination schedule preferences of the target population, vaccine hesitancy and fiscal space analysis (health sector's ability to accommodate the budget required for TCV introduction).

**Table 1:** Estimated priority scores for evidence factors supporting TCV decision-making in India

Rank	Evidence factors	Evidence gap score	Evidence importance score	Evidence (research) priority score
1	Perception of typhoid fever among the public	3.67	3.16	**6.83**
2	Budget requirement for TCV introduction	3.67	3.06	**6.73**
3	Vaccine (TCV) availability	3.67	2.69	**6.35**
4	Socioeconomic impact of typhoid fever	2.67	3.56	**6.23**
5	Fiscal space analysis	4.00	2.22	**6.22**
6	Antimicrobial resistance (AMR) tracking	2.33	3.70	**6.04**
7	Typhoid fever mortality	2.33	3.55	**5.89**
8	Perception of TCV among the public	3.67	2.04	**5.71**
9	Immunisation managers’ acceptance of TCV	3.33	2.04	**5.38**
10	Schedule preferences among parents	4.00	1.12	**5.12**
11	Cost-effectiveness among the underprivileged (equity)	3.00	2.10	**5.10**
12	Duration of vaccine (TCV) protection	2.00	2.92	**4.92**
13	Co-administration safety and immunogenicity	2.00	2.81	**4.81**
14	Vaccine (TCV) hesitancy	4.00	0.77	**4.77**
15	Severity of typhoid fever: complications and hospitalisations	1.00	3.75	**4.75**
16	Sustainable domestic funding for TCV	2.33	2.14	**4.47**
17	Public demand and willingness to pay for vaccines (TCV)	3.33	1.07	**4.41**
18	Incidence of typhoid fever	0.33	4.00	**4.33**
19	Robustness of typhoid fever surveillance system	3.00	1.32	**4.32**
20	Financial risk protection from vaccination	2.67	1.63	**4.30**
21	Increased health benefits from vaccination	1.33	2.65	**3.98**
22	Vaccine (TCV) efficacy	0.00	3.77	**3.77**
23	Population impact of vaccination	1.00	2.72	**3.72**
24	Acceptance among private medical practitioners	3.00	0.72	**3.72**
25	Ethical and cultural acceptability	3.33	0.30	**3.63**
26	Vaccine (TCV) safety	0.00	3.61	**3.61**
27	Feasibility of coadministration with other EPI vaccines	1.67	1.93	**3.60**
28	Regional/international considerations	0.33	3.19	**3.53**
29	Field effectiveness of vaccine (TCV)	0.00	3.38	**3.38**
30	Cost-effectiveness of vaccine (TCV)	0.00	3.35	**3.35**
31	Alternative typhoid fever control measures	0.00	3.34	**3.34**
32	National Technical Group on Immunisation in India (NTAGI) recommendation	0.00	3.28	**3.28**
33	Enhanced vaccine access from TCV	1.00	2.07	**3.07**
34	WHO recommendation on TCV	0.00	3.06	**3.06**
35	Public acceptability of vaccine (TCV)	2.00	0.92	**2.92**
36	External funding for TCV	0.67	1.60	**2.26**
37	Vaccine storage capacity	0.00	2.11	**2.11**
38	Vaccine characteristics	0.00	2.05	**2.05**
39	Human resources in the health system	0.00	1.90	**1.90**
40	Demonstration project on TCV for feasibility	1.33	0.46	**1.80**
41	Professional body (IAP) recommendation	0.00	1.72	**1.72**
42	Gavi/donor agency commitment	0.00	1.64	**1.64**
43	Vaccination coverage rate for EPI vaccines	0.00	1.10	**1.10**
44	Robustness of AEFI monitoring system	0.00	0.95	**0.95**
45	Robustness of Health Management Information System	0.00	0.00	**0.00**

Scores for the gap (0–4 scale), importance (0–4 scale) and priority (0–8 scale) of evidence for TCV decision-making in India (detailed scores of 45 evidence factors are available in online supplemental annex 6). Priority scores are the sum of gap and importance scores.

AEFIadverse event following immunisationGaviGavi, the vaccine allianceIAP-ACVIPIndian Academy of PaediatricsNTAGINational Technical Advisory Group on ImmunisationTCVtyphoid conjugate vaccine

### Stakeholder survey to assess perceived importance of evidence

Among seven EtR criteria, disease burden was ranked of high importance (RW=1.13/7) and equity of low importance (RW=5.96/7) by stakeholders for TCV decision-making in the Indian context (online supplemental annex 6). The top priorities under respective EtR criteria were typhoid fever incidence (RS=1.36/7; disease burden criteria), vaccine efficacy (RS=1.48/6; benefits and harms of the intervention criteria), disease perceptions among the target population (RS=1.22/6; values and preferences of the target population criteria), approval by NTAGI (RS=1.50/7; acceptability to stakeholders’ criteria), cost-effectiveness analysis (RS=1.42/5; resource use criteria), increased health benefits of TCV (RS=1.68/4; equity criteria) and sustainable TCV availability (RS=2.63/10; feasibility criteria). We multiplied respective RS and RW for each of the 45 evidence factors to estimate the ES and to derive the evidence IS (see for details) and presented it in table 1.

Based on evidence IS, the top five important evidence factors in decision-making from the stakeholder’s perspective were typhoid fever incidence, vaccine efficacy, typhoid fever severity, typhoid AMR tracking and vaccine safety (see table 1 and online supplemental annex 6).

Three participants also reported that manufacturing a vaccine within the country is crucial for vaccine introduction decisions in India, which was not on the list of EtR evidence factors.

All 22 participants responded to each of the three open-ended policy questions. For decision-making on the optimal age of TCV introduction—typhoid burden by age group, the number of vaccine doses in the immunisation schedule, caregivers’ perspective and vaccine efficacy by age group were the most important considerations among 15 factors (online supplemental annex 7). For decision-making on the state-wise roll-out of TCV—typhoid burden at the regional level (28 states and 8 union territories), human resource availability and political will were the most important considerations among 16 factors. For decision-making on additional school-based vaccination—typhoid burden in school age groups and logistic planning (feasibility) in school settings were the most important considerations among 20 factors.

### Defining evidence priorities

**Figure 3:**
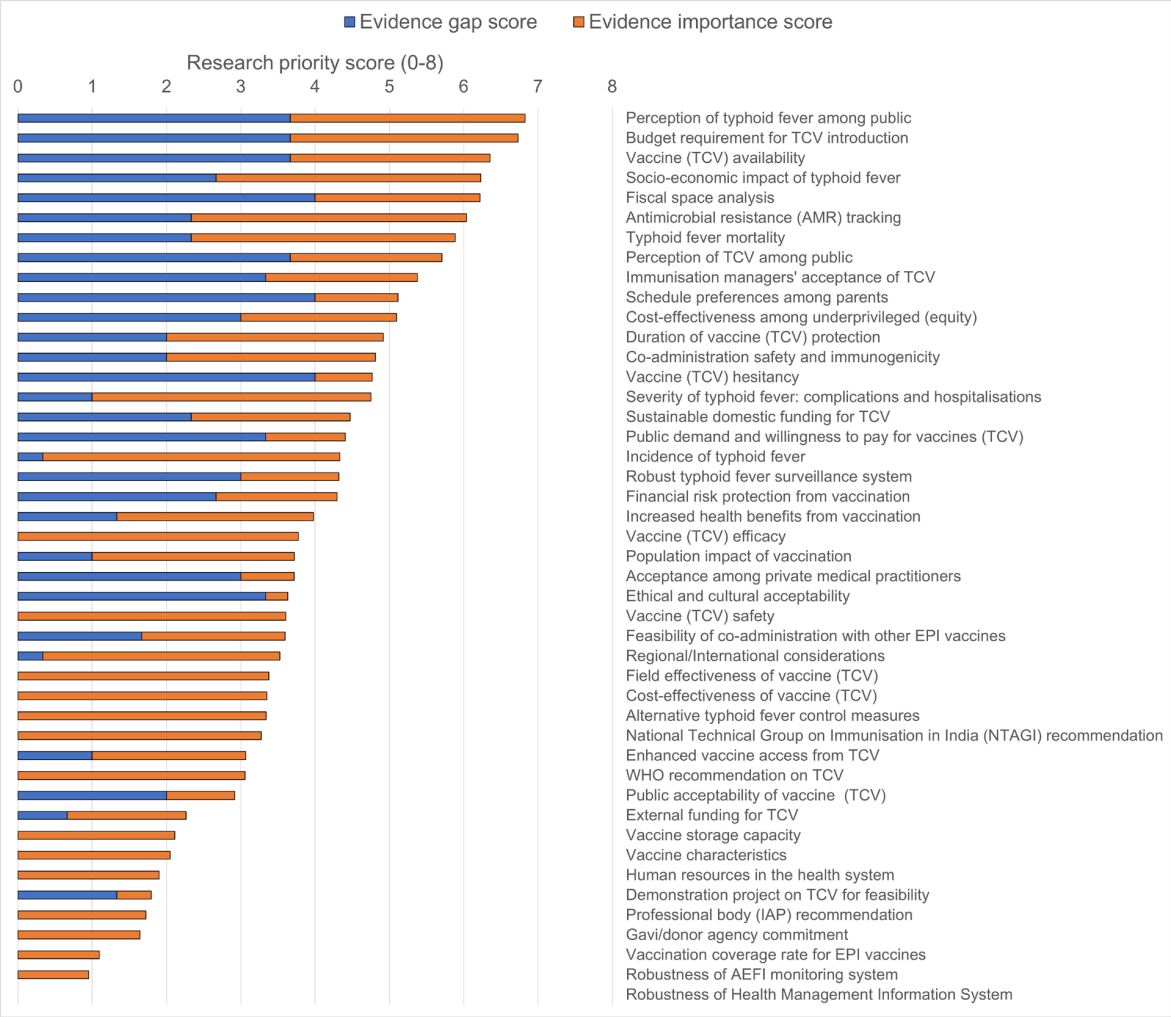
Evidence priorities. Evidence priority scores (scale of 0–8) were estimated by summing the evidence gap score (scale of 0–4) from the literature review and the evidence importance score (scale of 0–4) from the stakeholder survey. AEFI, adverse event following immunisation; IAP, Indian Academy of Paediatrics; TCV, typhoid conjugate vaccination.

We identified the top research priorities (on a scale of 0–8) to generate evidence for supporting TCV decision-making in the UIP of India by adding the scores for evidence gaps and stakeholders’ perceived importance. The top research priorities were disease perception among the target population (6.8), budget requirement for vaccination (6.7), sustainable vaccine availability (6.4), fiscal space analysis (6.2), the socioeconomic impact of typhoid fever (6.2), antimicrobial resistance tracking (6.0) and typhoid fever mortality (5.9) (see table 1, figure 3, online supplemental annex 6). As the subtle differences in evidence PS may not mean a real difference, we organised research priorities in five colour-coded groups of progressive priorities based on quintiles of the scores (online supplemental annex 8). We also presented 45 evidence factors in a 4 by four table comparing the evidence GS assessed from the literature with the perceived IS reported from the stakeholders’ survey (online supplemental annex 9). The evidence factors in the upper right-most corner of the table are of high gap and high importance, and the ones in the lower left-most corner are of low gap and low importance.

## Discussion

We adapted the WHO-EtR criteria and evidence factors to the Indian context. We assessed and compared evidence gaps to the key stakeholders’ perceived importance in developing a list of evidence/research priorities to support decision-making on TCV introduction and implementation in the UIP of India. We identified evidence priorities among three domains—acceptability to health workers and the public; financial and logistics considerations related to budget impact, vaccine supply and fiscal space; and the health and economic burden of typhoid fever reflected in socioeconomic burden, typhoid mortality and antimicrobial resistance tracking.

We demonstrated the applicability of the WHO-EtR framework at the country level to identify evidence priorities for decision-making on new vaccines beyond developing recommendations for a new vaccine introduction. We developed evidence factors aligned with the EtR framework and evaluated them in the Indian context. Specifically, we identified research priorities to generate evidence supporting decision-making on TCV in India. We addressed the need for countries to adapt to evidence factors and validated the EtR framework to support decision-making on new vaccines in national immunisation programmes. For example, vaccine availability within the country has emerged as an important consideration from the stakeholders’ perspective in supporting TCV decision-making in India. Recent vaccine introductions in India (rotavirus vaccine, pneumococcal conjugate vaccine and human papillomavirus vaccine) and coverage scale-up occurred once the vaccines manufactured within the country were available in India.^15 27 28^ As of 2023, four indigenous TCVs are available in India,^12^ which can help the implementation and scale-up of TCV.

Prioritising health interventions to achieve optimal health should be based on explicit and evidence-based criteria.^29^ The WHO approach to prioritisation considers data/evidence and dialogue with stakeholders as critical elements in decision-making.^30^ The decision-making process for introducing a new vaccine needs comprehensive evidence that takes substantial time and resources.^31^ Therefore, dialogue-based evidence prioritisation is necessary to optimise research resources for new vaccine introduction and implementation decisions. The existing tools and guidance on vaccine-related prioritisation that use dialogue-based approaches, such as a decision-support framework of the ‘Country-led Assessment for Prioritisation in Immunisation (CAPACITY)’,^32^ focus on prioritising or ranking multiple competing interventions in a structured approach. The WHO-EtR framework provides evidence criteria for decision-making^17 18^ but does not address evidence prioritisation. Our novel approach of combining country-adapted EtR framework-defined evidence with input from key stakeholders combines data with dialogue in evidence prioritisation to support decision-making on new vaccines. We used the principle of quantitative MCDA to compare evidence factors against each other. We developed a rank order of importance based on the stakeholders’ perspective by weighing and scoring different factors. Asking stakeholders to assign rank orders and multiplying rank orders of EtR criteria and evidence factors to obtain an ES is an efficient approach to elicit stakeholder perspectives. Our approach facilitates the reflection of the evidence priorities through rank order, reduces the cognitive burden on the stakeholders, reduces biases induced by dominant voices and improves the consistency and transparency of the decision-making process and outcomes. Our deductive approach to eliciting a final prioritisation list of evidence factors using literature and online anonymous dialogue minimises the subjective biases of the researchers and stakeholders.

Our study has limitations. First, assessing the strength of evidence is difficult to quantify precisely. The WHO-SAGE recommendation uses an international expert deliberation process to derive judgements on the quality of evidence. We used an approach to score three attributes of evidence (availability and sufficiency, quality, breadth and applicability) to minimise our subjective judgement bias. Second, our stakeholder survey includes sampling bias, non-response and response errors. We minimised these biases using purposive and snowball sampling, followed up with respondents individually through personal contacts, and offered extra support and guidance on technical challenges. Third, the stakeholders with specific subject expertise may have biases from their background and experience, which may influence the overall stakeholders’ perspective. We minimised this subjective bias by categorising stakeholders into 10 groups based on their role in the new vaccine introduction. We limited the maximum sample per group to four stakeholders to avoid undue influence of specific types of expertise. Fourth, rank-ordering evidence factors have intrinsic limitations because participants cannot place two evidence factors at the same rank. We explicitly mentioned this challenge during the survey. We asked the participants to make a note in the comment box so that we could rank two or more evidence factors at the same level during data analysis. Fifth, the stakeholders’ rank order of the seven EtR criteria was very influential to the final position of the 45 evidence factors. For example, there may be some evidence factors that were not considered important, but they have been elevated to a higher position simply because they belong to an EtR criterion that was ranked highly overall. Sixth, the stakeholder’s perceived importance data represent perceptions at the national level and does not capture regional variations. We need to obtain the perspective of state-level and district-level immunisation programme managers and health staff who implement the vaccination to determine the variations in the perceived importance of evidence factors across geographies in India. Seventh, the stakeholders’ perceptions associated with NTAGI may not fully represent the research priorities to support ongoing decision-making on TCVs in diverse Indian contexts and warrant future exploration. Finally, the evidence requirements and priorities evolve dynamically with decision stages. Therefore, the research priorities presented here need future updates and should advance along with the decision stage, time and availability of new evidence. Understanding this dynamic prioritisation in the country context is necessary before acting on evidence and research needs.

In summary, we combined evidence gaps to the stakeholders’ perceived importance in generating research/evidence priorities for supporting TCV’s ongoing decision-making in the UIP of India. The key research priorities for decision-making on TCV in India are acceptability to immunisation managers/health staff and the public, financial considerations related to budget impact and fiscal space, and the health and economic burden of typhoid fever represented by socioeconomic impact, mortality and antimicrobial resistance tracking. Evidence on state-wise and age-wise typhoid incidence, vaccine efficacy by age group, number of vaccine doses (coadministration) in the immunisation schedule, caregivers’ perspectives, operational feasibility and human resources are other key considerations for developing TCV implementation strategies in India. We recommend that future research focus on the disease, mortality and economic burdens of typhoid fever related to antimicrobial resistance, TCV acceptability to health staff and the public and exploring the budget impact and financing of TCV introduction in India. The broad impact of our study is that for any new vaccine introduction in any country, our novel method to identify research priorities can be applied to generate evidence for supporting decision-making in national immunisation programmes.

## supplementary material

10.1136/bmjph-2024-001089 online supplemental file 1

10.1136/bmjph-2024-001089 online supplemental file 2

10.1136/bmjph-2024-001089 online supplemental file 3

## Data Availability

All data relevant to the study are included in the article or uploaded as online supplemental information.
