# Supplementary material for: Research priorities to support typhoid conjugate vaccine decision-making in India: evidence assessment and stakeholder survey
Source: BMJ Public Health. 2024 Oct 3;2(2):e001089. doi: 10.1136/bmjph-2024-001089 (PMC11816874; doi:10.1136/bmjph-2024-001089)
Supplement: online supplemental file 1 [file bmjph-2-2-s001.pdf]

Research priorities to support typhoid conjugate vaccine decision-making in India: evidence assessment and stakeholder survey

Vijayalaxmi V Mogasale[1,2], Anish Sinha[3], Jacob John[4], Habib Hasan Farooqui[5], Arindam Ray[6], Tracey Chantler[7], Vittal Mogasale[8], Bhim Gopal Dhoubhadel[2,9,†], W John Edmunds[1,2,†], Andrew Clark[10,†], Kaja Abbas[1,2,11,†]*

[1]*Department of Infectious Disease Epidemiology and Dynamics, London School of Hygiene & Tropical Medicine, London, UK*

[2]*School of Tropical Medicine and Global Health, Nagasaki University, Japan*

[3]*Indian Institute of Public Health-Gandhinagar, India*

[4]*Department of Community Health & Development, Christian Medical College, Vellore, India*

[5]*College of Medicine, Qatar University, Doha, Qatar*

[6]*Department of Infectious Disease & Vaccine Delivery, Bill and Melinda Gates Foundation, New Delhi, India*

[7]*Department of Global Health and Development, London School of Hygiene & Tropical Medicine, London, UK*

[8]*Graduate School of Public Health Yonsei University, Seoul, Republic of Korea. (Current affiliation: Health Financing and Economics Department, World Health Organisation, Geneva, Switzerland)*

[9]*Institute of Tropical Medicine, Nagasaki University, Nagasaki, Japan*

[10]*Department of Health Services Research and Policy, London School of Hygiene & Tropical Medicine, London, UK*

[11]*Public Health Foundation of India, New Delhi, India*

[†]*Share senior authorship*

Correspondence: Vijayalaxmi V Mogasale, Department of Infectious Disease Epidemiology and Dynamics, London School of Hygiene & Tropical Medicine, London, UK. Email: vijayalaxmi.mogasale@lshtm.ac.uk

Annexes

Annex 1. Literature review, World Health Organisation (WHO) Evidence-to-Recommendation (EtR) framework [16,17] and India-adapted EtR evidence factors to support TCV decision-making in India

a. Methodology of literature review and number of publications included in the review

We conducted a literature review to identify and summarise Indian data relevant to each of the seven criteria in the WHO-EtR framework. The search included PubMed (search 1: "typhoid*" and "India"; search 2: "typhoid conjugate vaccine"), WHO The Strategic Advisory Group of Experts on Immunisation (SAGE) background documents, grey literature specific to India, and personal contact with researchers working in the area of typhoid fever in India. Our summary includes available evidence until 30th November 2023 with no limit on the search start date. No language limitation included.

The PubMed search yielded 1625 and 130 results from searches 1 and 2, respectively. In addition, WHO-SAGE meeting background documents, Indian NTAGI meeting minutes, clinical trial registry, and coalition against typhoid fever reports were reviewed. The evidence to support further TCV decision-making in India was summarised and presented under seven WHO-EtR criteria, and the number of publications identified by criteria is listed below.

WHO-EtR Criteria	Number of Peer-reviewed journal publications included*	Other literatures included
Problem	20	0
Benefits and harms of the intervention	19	3 (clinical trial registries)
Values and preferences of the target population	2	0
Acceptability to stakeholders	5	1 NTAGI meeting minute, 2 websites of international organisations
Resources use	7	0
Equity	2	0
Feasibility	10	5 Websites of ministry/international organisations

*Some publications were used for more than 1 EtR criterion, and therefore, numbers cannot be totalled.

b. WHO-EtR criteria, WHO-EtR evidence factors and India adapted EtR evidence factors

WHO-EtR Criteria	WHO-EtR evidence factors	India-adapted EtR evidence factors*
1. Problem	1. Burden/epidemiology of the disease/severity/social impact<br>2. Clinical characteristics of the disease (sign, symptoms and complications)<br>3. Use and Costs of Health Care<br>4. Alternative preventive and control measures<br>5. Regional and international considerations	1. Typhoid incidence<br>2. Disease severity<br>3. Mortality<br>4. Antimicrobial resistance (AMR)<br>5. Socio-economic impact/health care costs<br>6. Alternative typhoid control measures<br>7. Regional/International considerations
2. Benefits and harms of the intervention	6. Efficacy and effectiveness of the intervention (benefits)<br>7. Safety of the intervention (harms)<br>8. Indirect effects of the intervention	8. Efficacy<br>9. Field effectiveness<br>10. Safety<br>11. Co-administration safety and immunogenicity<br>12. Duration of protection<br>13. Population impact
3. Values and preferences of the target population	9. Perception of the target population of the intervention and the disease<br>10. Differences by subgroups of target population<br>11. Demand	14. Disease perception<br>15. Perception of TCV<br>16. Ethical and cultural acceptability<br>17. Demand and willingness to pay for vaccines<br>18. Schedule preferences<br>19. Vaccine hesitancy

4. Acceptability to stakeholders	12. Acceptability of the intervention<br>13. Financial, ethical and programmatic considerations	20. Acceptability to WHO<br>21. Acceptability to Gavi/donor agencies<br>22. Acceptability to NTAGI & MOH<br>23. Acceptability to Professional bodies<br>24. Acceptability to Immunisation programme managers<br>25. Acceptability to Private medical practitioners<br>26. Public acceptability
5. Resources use	14. Resource use and size of resource requirement<br>15. Cost-effectiveness<br>16. Reasonable/ efficient allocation<br>17. Economic impact of the intervention on immunization programme and health sector	27. Cost-effectiveness<br>28. Budget requirement<br>29. Sustainable funding<br>30. Fiscal space analysis<br>31. External funding
6. Equity	18. Access to intervention<br>19. Ethics, legality of the intervention<br>20. Stigma	32. Increased health benefits<br>33. Financial risk protection<br>34. Enhanced vaccine access<br>35. Cost-effectiveness/value for poor
7. Feasibility	21. Vaccine characteristics<br>22. Accessibility<br>23. Resources for storage, distribution<br>24. Information management<br>25. Disease and AEFI surveillance<br>26. Global, regional, local experiences<br>27. Vaccine availability	36. Human resources<br>37. Vaccine storage capacity<br>38. AEFI monitoring system<br>39. Health Management Information System<br>40. Infectious disease surveillance system<br>41. Vaccine characteristics<br>42. Vaccine availability<br>43. EPI vaccination coverage<br>44. Co-administration feasibility<br>45. Feasibility through demonstration project

*We defined India-adapted evidence factors in congruent with public health system in India and conforming meeting minutes of the India's National Technical Advisory Group on Immunisation (NTAGI), which describes different evidence discussed before making decisions. We developed India-adapted evidence factors by dividing evidence list under WHO-EtR criteria. For example, AMR was included based on NTAGI meeting minutes, whereas "Burden/epidemiology of the disease/severity" was broken into three evidence factors: "typhoid incidence, typhoid severity, and mortality".

Annex 2: Literature assessment showing the strength of evidence scores for India-adapted evidence factors

The WHO- Evidence to Recommendation (EtR) criteria and evidence factors [1] strength of evidence scoring table

A score for each evidence factor was given between 0 to 4 based on the below-described criteria for each of the four attributes of the evidence. The score attributes were modified and adapted from "Systems to Rate the Strength Of Scientific Evidence" [2,3]. The scoring definition corresponds to the GRADE scoring approach [4,5]. As the GRADE approach was not applicable to the availability and breadth questions and could not be used for some evidence factors, we used a scoring system with 0-4, with 1-4 mapping to GRADE levels of certainty, and an additional option (0) added to indicate where evidence is unavailable.

- Score 4: We are very confident that the true effect lies close to that of the estimate of the effect on health outcomes.
- Score 3: We are moderately confident in the estimate of the effect on health outcomes. The true effect is likely to be close to the estimate of the effect.
- Score 2: Our confidence in estimating the effect on the health outcome is limited.
- Score 1: We have very little confidence in estimating the effect on the health outcome.
- Score 0: No data was found during the literature assessment

The description of questions:

- Is the evidence available and sufficient? This question looks into the **availability and sufficiency** of the number of studies/data points, number of sites, sample size or power.
- Is the **quality of the evidence** acceptable? This question looks into study design, limitations, inconsistencies, imprecisions, biases, effect size /strength of association and confounding.
- Does the **breadth of evidence** cover all dimensions of evidence under consideration? This question looks into the dimensions and applicability of evidence in developing TCV implementation strategies, including age distribution, evidence on school age groups, urban/rural differences, and state-wise distribution.

	**EtR Criteria and Evidence Factors**	**Strength of evidence-scoring questions**			**Key highlights used in scoring**	References
	**Disease burden**	Evidence availability and sufficiency	The quality of the evidence	The breadth of evidence	Evidence (Limitations, inconsistencies, indirectness, imprecisions, biases, large effect/strength of association, population effect, confounding, study design, context, applicability)	
1	**Typhoid incidence:** Is the typhoid incidence in India high enough to consider it a priority disease?	4	4	3	Multi-site, urban-rural, prospective surveillance data is available and showed high incidence. State-level estimates are done based on modelling. Several site-specific and age-specific incidence data have been published in the last two decades.	[6-12]
2	**Regional/International considerations:** Is typhoid burden high in neighbouring and other low- and middle-income countries with a potential to spread make it a priority in India?	4	4	3	Several global and regional studies have shown a high burden of typhoid fever in Neighbouring countries as well as other LMICs. WHO has summarised it in a vaccine recommendation paper.	[13-16]
3	**AMR:** As antimicrobial resistance (AMR) in *S.* Typhi limits treatment options and increases its potential for spread, is tracking AMR a priority in India?	1	3	1	A systematic review of AMR in S. Typhi showed data from selected sites. A hospital-based AMR surveillance system is available. State specific and age specific data at national scale was not found.	[17,18]

4	**Mortality:** Is typhoid-related death high in the Indian population?	2	1	2	Limited estimates of mortality are available from multi-site surveillance. Data is limited on state-wise, age-wise, urban/rural classification. Variable healthcare access is likely to influence mortality.	19
5	**Socio-economic impact:** Are the health care costs to the government and households and loss of workdays due to typhoid high in India?	1	2	1	There are cost of illness studies available. There is a limited description of the socioeconomic impact, including catastrophic expenditures.	20-23
6	**Alternative typhoid control measures:** Are health education, improving hygiene, and food handling practices practical, effective and affordable in India?	4	4	4	Alternative control measures for typhoid fever are well known in the literature, as documented in a vaccine position paper.	16,24
7	**Disease severity:** Are typhoid complications and hospitalisation rates high in the Indian population?	3	3	3	The disease severity is documented in the multisite surveillance data. There are some publications from the past. However, age-wise and state-wise documentation is limited.	6,25
	**Safety and efficacy**					
8	**Efficacy:** Does TCV show good efficacy based on clinical trials?	4	4	4	There are several studies inside and outside India that demonstrated the efficacy of TCV.	26-30
9	**Safety:** Is TCV shown to be safe based on clinical trials?	4	4	4	There are several studies inside and outside India that demonstrated the safety of TCV.	1,26,28-30
10	**Field effectiveness:** Does the demonstration project show TCV is safe and effective in field situations?	4	4	4	There are several studies inside and outside India that demonstrated the field effectiveness of TCV.	31-34
11	**Co-administration safety and immunogenicity:** Is TCV co-administration with other EPI vaccines (e.g., MR, IPV, JE, DPT) safe and immunogenic?	1	3	2	Studies outside India, including in Nepal, have demonstrated safety and immunogenicity of co-administration. Some vaccines in Indian UIP, such as JE, IPV and pneumococcal vaccine co-administration safety and immunogenicity are not demonstrated.	35 36 37
12	**Duration of protection:** Does TCV offer long-term protection?	2	3	1	TCV is generally believed to protect long-term. There is limited data in Indian and worldwide settings.	16,27,38
13	**Population impact:** Does TCV introduction have a large protective effect (including herd protection) at the population level to control typhoid?	2	4	3	Modelling studies have demonstrated population-level impact. Post-vaccination evaluations outside India demonstrated population benefits. Data from field settings are limited.	39-42
	**Values and preferences of the target population**					
14	**Disease perception:** How does the target population feel the risk of getting typhoid fever among their children to consider the vaccination?	1	0	0	No studies were found on the values and preferences of the target population related to typhoid fever. However, a study in 2009 assessed disease perceptions in clinical trial context.	43
15	**Perception of TCV:** How does the target population value or perceive the desirable and undesirable effects of the TCV introduction?	0	0	1	No studies were found on the values and preferences of the target population related to TCV. However, TCV is available and used in private market.	43,44
16	**Ethical and cultural acceptability:** Does the target population perceive TCV as an acceptable intervention ethically and culturally?	0	0	2	No studies were found on the values and preferences of the target population related to ethical and cultural acceptability. However, vaccines are generally well accepted by the target population.	

17	**Demand and willingness to pay for vaccines:** How much money are parents willing to pay for TCV, and when introduced in the routine immunisation program, will there be demand?	0	0	2	No studies were found on the values and preferences of the target population related to demand and willingness to pay for TCV. However, in general there is good demand for vaccines under universal immunisation programme	
18	**Schedule preferences:** Considering multiple injections under the EPI schedule, do parents prefer TCV at 9-12 months or 16-24 months?	0	0	0	No studies were found on the values and preferences of the target population related to TCV schedule preferences.	
19	**Vaccine hesitancy:** Does target population perception indicate a potential risk for TCV hesitancy?	0	0	0	No studies were found on the values and preferences of the target population related to TCV hesitancy.	
	**Acceptability to stakeholders**					
20	**WHO:** Is TCV recommended by WHO?	4	NA	NA	WHO has issued a TCV recommendation paper demonstrating acceptability.	[16]
21	**Gavi/donor agency:** Has Gavi included TCV in its vaccine portfolio? Are donors ready to support TCV introduction in India?	4	NA	NA	Gavi included TCV in their portfolio demonstrating acceptability.	[45,46]
22	**NTAGI:** Is the National Technical Group on Immunisation in India (NTAGI) recommended TCV introduction in India?	4	NA	NA	NTAGI has issued a TCV introduction recommendation demonstrating acceptability.	[47]
23	**Professional body:** Is professional body like Indian Academy Paediatrics (IAP) recommended TCV use?	4	NA	NA	IAP-ACVIP has issued a TCV use recommendation demonstrating acceptability.	[48]
24	**Immunisation managers:** Do immunisation managers accept TCV as an additional vaccine in the routine immunisation schedule?	0	0	2	The acceptance by immunisation service providers in public sector was not found. But immunization managers generally accept new vaccines that are part of UIP.	
25	**Private medical practitioners:** Is TCV valued by private medical practitioners and accepted in the private market?	0	0	3	The data on acceptance by immunisation service providers in the private sector was not found. However, TCV is available and used in the private market.	[44]
26	**Public acceptability:** Does the demonstration project show the programmatic and public acceptability of TCV?	2	2	2	The public acceptability of TCV is demonstrated on a small scale in the Navi Mumbai TCV introduction project. TCV is available and used in the private sector with no reliable coverage estimates.	[49]
	**Resource requirement and value for money (cost-effectiveness)**					
27	**Cost-effectiveness:** Is TCV introduction in routine immunisation in India a good value for money?	4	4	4	Many cost-effectiveness studies are available for Indian settings as well as for other settings.	[39-42]
28	**Budget requirement:** How much money is needed for the routine introduction of TCV in India? Is it large?	0	0	1	No study was found on budget requirements for TCV introduction. However, an estimation of TCV delivery costs is available for Navi Mumbai.	[50]
29	**Sustainable funding:** Is there a domestic (government) financial commitment that can sustainably support TCV in the future?	1	1	3	Several new vaccine introductions in recent years show financial commitment from the government for sustained financing.	[51-53]

30	**Fiscal space analysis:** Does the health budget in India has space to accommodate the budget of introducing TCV? In other words, is there enough money for the TCV introduction?	0	0	0	No study was found on fiscal space analysis or immunisation budget impact of the introduction of TCV in UIP.	
31	**External funding:** Are Gavi or other donors willing to fund TCV implementation in India?	3	3	4	Gavi has included TCV in the portfolio and committed funding to India. BMGF has funded large-scale typhoid-related research studies in India.	[54,55]
	**Equity aspect**					
32	**Increased health benefits:** Does TCV introduction offer improve health benefits to the poor, vulnerable, or people living in urban slums?	3	3	2	No study was found on equity analysis demonstrating improved health benefits to the poor. But generally, vaccination is known to increase health benefits in the poor.	
33	**Financial risk protection:** Does TCV introduction decreases catastrophic health care expenses and protect from financial risks in the population?	0	2	2	No study on equity analysis demonstrating financial risk protection was found. However, COI studies have shown the catastrophic expenses caused by typhoid fever.	[20]
34	**Enhanced vaccine access:** Does the routine introduction of TCV improve current TCV coverage in poor, vulnerable, or people living in urban slums, as now it is limited to the private market?	3	3	3	No study was found on equity analysis demonstrating enhanced vaccine access. However, in principle, the introduction of TCV in UIP enhances vaccine access.	
35	**Cost-effective in poor:** Is TCV introduction a good value for money for the poor, vulnerable, or people living in urban slums?	1	1	1	No study was found on equity analysis demonstrating cost-effectiveness to the poor. Cost-effectiveness of urban/rural vaccination is available.	
	**Feasibility of vaccination**					
36	**Human resources:** Are the available human resources sufficient?	4	4	4	The capacity of human resources is demonstrated by several new vaccine introductions, including an MR campaign that targeted 400 million children and other new vaccine introductions.	[51-53,56]
37	**Vaccine storage capacity:** Is available cold chain capacity sufficient?	4	4	4	The immunisation system in India has a vast vaccine delivery network of more than 27,000 functional cold-chain points under the digitally monitored Vaccine Intelligence Network (eVIN).	[57,58]
38	**AEFI monitoring:** Is the Adverse Events Following Immunisation system robust enough to track post-introduction TCV AEFI?	4	4	4	India has a well-established Adverse Events Following Immunisation (AEFI) program with guidelines for monitoring and reporting AEFI.	[59,60]
39	**HMIS:** Is Health Management Information System robust enough to track coverage and utilisation of TCV?	4	4	4	India has a well-established Health Management Information System (HMIS) through various levels of immunisation systems.	
40	**Infectious disease surveillance system:** Is the surveillance system robust enough to monitor post-TCV introduction typhoid cases?	1	1	1	India has established a good Integrated Disease Surveillance Program (IDSP) to conduct disease surveillance for infectious diseases to detect and respond to outbreaks immediately. However, typhoid diagnosis is mainly done presumptively (clinically) without blood culture confirmation, which needs to be strengthened.	[61]

41	**Vaccine characteristics:** Does TCV presentation and route of administration make it feasible to deliver the vaccine efficiently by service providers?	4	4	4	There are four licensed typhoid conjugate vaccines (TCV) available in India, of which two are WHO-prequalified. They are suitable for UIP in multidose vials, as used in the Navi Mumbai demonstration project.	[49,62]
42	**Vaccine availability:** Is there sustainable supply of TCV in India?	0	0	2	A large-scale introduction in India needs a good forecast of demand and supply and a vaccine procurement plan, which was not available. However, there are 4 indigenous vaccine manufacturers which have a large capacity for vaccine production. Large amounts of TCV produced are committed for export.	
43	**Vaccination coverage**: Is current EPI vaccine coverage high enough to accommodate the additional load of TCV introduction?	4	4	4	The capacity to deliver vaccine is demonstrated by high coverage of DTP ranging from 82% to 91% and MCV1 coverage from 83% to 95% from 2012 to 2021, as reported by UNICEF.	[63]
44	**Co-administration:** Is it feasible to co-administer TCV with other EPI vaccines (e.g., DPT, MR, IPV, JE)?	2	2	3	The TCV can fit easily into UIP but will increase the number of injections given in each immunisation visit. No data was available on the operational feasibility of co-administration.	
45	**Demonstration project:** Is the feasibility of TCV implementation established in field settings?	2	4	2	The feasibility of TCV implementation is demonstrated on a small scale in the Navi Mumbai TCV introduction project. However, it is not studied in other Indian settings.	[49]

References

1. WHO. Evidence to Recommendation table- Typhoid Vaccines. World Health Organization. https://cdn.who.int/media/docs/default-source/immunization/position_paper_documents/typhoid/6-sage-typhoid-e2r-final.pdf?sfvrsn=fcaa83ec_2 (accessed.
2. West S, King V, Carey TS, et al. Systems to rate the strength of scientific evidence. *Evid Rep Technol Assess (Summ)* 2002; (47): 1-11.
3. Lohr KN. Rating the strength of scientific evidence: relevance for quality improvement programs. *Int J Qual Health Care* 2004; **16**(1): 9-18.
4. Balshem H, Helfand M, Schünemann HJ, et al. GRADE guidelines: 3. Rating the quality of evidence. *J Clin Epidemiol* 2011; **64**(4): 401-6.
5. Duclos P, Durrheim DN, Reingold AL, Bhutta ZA, Vannice K, Rees H. Developing evidence-based immunization recommendations and GRADE. *Vaccine* 2012; **31**(1): 12-9.
6. John J, Bavdekar A, Rongsen-Chandola T, et al. Burden of Typhoid and Paratyphoid Fever in India. *N Engl J Med* 2023; **388**(16): 1491-500.
7. Cao Y, Karthikeyan AS, Ramanujam K, et al. Geographic Pattern of Typhoid Fever in India: A Model-Based Estimate of Cohort and Surveillance Data. *J Infect Dis* 2021; **224**(224 Supple 5): S475-S83.
8. Sinha B, Rongsen-Chandola T, Goyal N, et al. Incidence of Enteric Fever in a Pediatric Cohort in North India: Comparison with Estimates from 20 Years Earlier. *J Infect Dis* 2021; **224**(Supple 5): S558-S67.
9. John J, Van Aart CJ, Grassly NC. The Burden of Typhoid and Paratyphoid in India: Systematic Review and Meta-analysis. *PLoS Negl Trop Dis* 2016; **10**(4): e0004616.
10. Ochiai RL, Acosta CJ, Danovaro-Holliday MC, et al. A study of typhoid fever in five Asian countries: disease burden and implications for controls. *Bull World Health Organ* 2008; **86**(4): 260-8.
11. Sinha A, Sazawal S, Kumar R, et al. Typhoid fever in children aged less than 5 years. *Lancet* 1999; **354**(9180): 734-7.
12. Sur D, Ochiai RL, Bhattacharya SK, et al. A cluster-randomized effectiveness trial of Vi typhoid vaccine in India. *N Engl J Med* 2009; **361**(4): 335-44.
13. Collaborators GTaP. The global burden of typhoid and paratyphoid fevers: a systematic analysis for the Global Burden of Disease Study 2017. *Lancet Infect Dis* 2019; **19**(4): 369-81.
14. Antillón M, Warren JL, Crawford FW, et al. The burden of typhoid fever in low- and middle-income countries: A meta-regression approach. *PLoS Negl Trop Dis* 2017; **11**(2): e0005376.
15. Mogasale V, Maskery B, Ochiai RL, et al. Burden of typhoid fever in low-income and middle-income countries: a systematic, literature-based update with risk-factor adjustment. *Lancet Glob Health* 2014; **2**(10): e570-80.
16. World Health Organization. Typhoid vaccines: WHO position paper, March 2018 - Recommendations. *Vaccine* 2019; **37**(2): 214-6.
17. Britto CD, John J, Verghese VP, Pollard AJ. A systematic review of antimicrobial resistance of typhoidal. *Indian J Med Res* 2019; **149**(2): 151-63.
18. Kaur J, Dhama AS, Buttolia H, et al. ICMR's Antimicrobial Resistance Surveillance system (*JAC Antimicrob Resist* 2021; **3**(1): dlab023.
19. Samuel P, Njarekkattuvalappil SK, Kumar D, et al. Case-Fatality Ratio of Enteric Fever: Estimates From Multitiered Surveillance in India. *J Infect Dis* 2021; **224**(Supple 5): S517-S21.
20. Kumar D, Sharma A, Rana SK, et al. Cost of Illness Due to Severe Enteric Fever in India. *J Infect Dis* 2021; **224**(Supple 5): S540-S7.
21. Poulos C, Riewpaiboon A, Stewart JF, et al. Cost of illness due to typhoid fever in five Asian countries. *Trop Med Int Health* 2011; **16**(3): 314-23.
22. Sur D, Chatterjee S, Riewpaiboon A, Manna B, Kanungo S, Bhattacharya SK. Treatment cost for typhoid fever at two hospitals in Kolkata, India. *J Health Popul Nutr* 2009; **27**(6): 725-32.
23. Bahl R, Sinha A, Poulos C, et al. Costs of illness due to typhoid fever in an Indian urban slum community: implications for vaccination policy. *J Health Popul Nutr* 2004; **22**(3): 304-10.
24. Im J, Islam MT, Ahmmed F, et al. Can Existing Improvements of Water, Sanitation, and Hygiene (WASH) in Urban Slums Reduce the Burden of Typhoid Fever in These Settings? *Clin Infect Dis* 2021; **72**(11): e720-e6.
25. Srinivasan M, Sindhu KN, Nag A, et al. Hospitalization Rates and Direct Medical Costs for Fever in a Pediatric Cohort in South India. *J Infect Dis* 2021; **224**(Supple 5): S548-S57.
26. Kundu R, Kandulna AK, Nayak U, et al. Immunogenicity and Safety of Typhoid Conjugate Vaccine in Healthy Indian Subjects: A Randomized, Active-controlled, Comparative Clinical Trial. *Indian Pediatr* 2020; **57**(7): 625-30.

27. Kandulna AK, Uttam KG, Sharma S, et al. Long-term Persistence of Immunogenicity After Primary Vaccination and Response to Booster Vaccination With Typhoid Conjugate Vaccine: Results of a Phase IV Extension Study. *Indian Pediatr* 2022; **59**(5): 388-92.
28. Shakya M, Colin-Jones R, Theiss-Nyland K, et al. Phase 3 Efficacy Analysis of a Typhoid Conjugate Vaccine Trial in Nepal. *N Engl J Med* 2019; **381**(23): 2209-18.
29. Patel PD, Patel P, Liang Y, et al. Safety and Efficacy of a Typhoid Conjugate Vaccine in Malawian Children. *N Engl J Med* 2021; **385**(12): 1104-15.
30. Qadri F, Khanam F, Liu X, et al. Protection by vaccination of children against typhoid fever with a Vi-tetanus toxoid conjugate vaccine in urban Bangladesh: a cluster-randomised trial. *Lancet* 2021; **398**(10301): 675-84.
31. Hoffman SA, LeBoa C, Date K, et al. Programmatic Effectiveness of a Pediatric Typhoid Conjugate Vaccine Campaign in Navi Mumbai, India. *Clin Infect Dis* 2023.
32. Yousafzai MT, Karim S, Qureshi S, et al. Effectiveness of typhoid conjugate vaccine against culture-confirmed Salmonella enterica serotype Typhi in an extensively drug-resistant outbreak setting of Hyderabad, Pakistan: a cohort study. *Lancet Glob Health* 2021; **9**(8): e1154-e62.
33. Thobani RS, Yousafzai MT, Sultana S, et al. Field evaluation of typhoid conjugate vaccine in a catch-up campaign among children aged 9 months to 15 years in Sindh, Pakistan. *Vaccine* 2022; **40**(36): 5391-8.
34. Batool R, Tahir Yousafzai M, Qureshi S, et al. Effectiveness of typhoid conjugate vaccine against culture-confirmed typhoid in a peri-urban setting in Karachi: A case-control study. *Vaccine* 2021; **39**(40): 5858-65.
35. Sirima SB, Ouedraogo A, Barry N, et al. Safety and immunogenicity of co-administration of meningococcal type A and measles-rubella vaccines with typhoid conjugate vaccine in children aged 15-23 months in Burkina Faso. *Int J Infect Dis* 2021; **102**: 517-23.
36. Sirima SB, Ouedraogo A, Barry N, et al. Safety and immunogenicity of Vi-typhoid conjugate vaccine co-administration with routine 9-month vaccination in Burkina Faso: A randomized controlled phase 2 trial. *Int J Infect Dis* 2021; **108**: 465-72.
37. Saluja T, Rai GK, Chaudhary S, et al. Immune non-interference and safety study of Vi-DT typhoid conjugate vaccine with a measles, mumps and rubella containing vaccine in 9-15 months old Nepalese infants. *Vaccine* 2022; **40**(40): 5828-34.
38. Vadrevu KM, Raju D, Rani S, et al. Persisting antibody responses to Vi polysaccharide-tetanus toxoid conjugate (Typbar TCV®) vaccine up to 7 years following primary vaccination of children < 2 years of age with, or without, a booster vaccination. *Vaccine* 2021; **39**(45): 6682-90.
39. Antillón M, Bilcke J, Paltiel AD, Pitzer VE. Cost-effectiveness analysis of typhoid conjugate vaccines in five endemic low- and middle-income settings. *Vaccine* 2017; **35**(27): 3506-14.
40. Burrows H, Antillón M, Gauld JS, et al. Comparison of model predictions of typhoid conjugate vaccine public health impact and cost-effectiveness. *Vaccine* 2023; **41**(4): 965-75.
41. Chauhan AS, Kapoor I, Rana SK, et al. Cost effectiveness of typhoid vaccination in India. *Vaccine* 2021; **39**(30): 4089-98.
42. Ryckman T, Karthikeyan AS, Kumar D, et al. Comparison of Strategies for Typhoid Conjugate Vaccine Introduction in India: A Cost-Effectiveness Modeling Study. *J Infect Dis* 2021; **224**(Supple 5): S612-S24.
43. Sur D, Manna B, Chakrabarty N, et al. Vaccine desirability during an effectiveness trial of the typhoid fever polysaccharide Vi vaccine in Kolkata India. *Hum Vaccin* 2009; **5**(9): 614-20.
44. Farooqui HH, Zodpey S. Private sector vaccine share in overall immunization coverage in India: Evidence from private sector vaccine utilization data (2012-2015). *Indian J Public Health* 2020; **64**(1): 75-8.
45. Neuzil K. More typhoid conjugate vaccines, more impact2020. https://www.coalitionagainsttyphoid.org/moretyphoidconjugatevaccines/ (accessed.
46. Gavi. New typhoid vaccine to receive Gavi support. 2018. https://www.gavi.org/news/media-room/new-typhoid-vaccine-receive-gavi-support (accessed.
47. GOI. Minutes of the meeting of 17th National Technical Advisory Group on Immunization (NTAGI), held on 28th June 2022, under the Chairpersonship of Secretary Family Welfare) at Nirman Bhawan, New Delhi2022. https://main.mohfw.gov.in/sites/default/files/17th%20NTAGI%20Meeting%20Minutes%20June%2028%2C%202022.pdf (accessed.
48. Vashishtha VM, Kalra A, Bose A, et al. Indian Academy of Pediatrics (IAP) recommended immunization schedule for children aged 0 through 18 years, India, 2013 and updates on immunization. *Indian Pediatr* 2013; **50**(12): 1095-108.
49. Date K, Shimpi R, Luby S, et al. Decision Making and Implementation of the First Public Sector Introduction of Typhoid Conjugate Vaccine-Navi Mumbai, India, 2018. *Clin Infect Dis* 2020; **71**(Suppl 2): S172-S8.

50. Song D PS, Shimpi R, Ramaswamy N, Haldar P, Harvey P, et al. Delivery cost of the first public sector introduction of typhoid conjugate vaccine in Navi Mumbai, India. *PLOS Glob Public Health* 2023; **3**(1): e0001396.
51. Malik A, Haldar P, Ray A, et al. Introducing rotavirus vaccine in the Universal Immunization Programme in India: From evidence to policy to implementation. *Vaccine* 2019; **37**(39): 5817-24.
52. Gupta SK, Sosler S, Lahariya C. Introduction of Haemophilus influenzae type b (Hib) as pentavalent(DPT-HepB-Hib) vaccine in two states of India. *Indian Pediatr* 2012; **49**(9): 707-9.
53. Varghese R, Veeraraghavan B, Jeyaraman Y, Kumar G, Arora NK, Balasubramanian S. Pneumococcal conjugate vaccine rollout in India: Expectations and challenges. *Indian J Med Microbiol* 2019; **37**(2): 141-6.
54. GAVI. India. Gavi support.2023. https://www.gavi.org/programmes-impact/country-hub/south-east-asia/india (accessed.
55. Carey ME, MacWright WR, Im J, et al. The Surveillance for Enteric Fever in Asia Project (SEAP), Severe Typhoid Fever Surveillance in Africa (SETA), Surveillance of Enteric Fever in India (SEFI), and Strategic Typhoid Alliance Across Africa and Asia (STRATAA) Population-based Enteric Fever Studies: A Review of Methodological Similarities and Differences. *Clin Infect Dis* 2020; **71**(Suppl 2): S102-S10.
56. Chatterjee S, Song D, Das P, et al. Cost of conducting Measles-Rubella vaccination campaign in India. *Hum Vaccin Immunother* 2022; **18**(1): 1-8.
57. Gurnani V, Dhalaria P, Chatterjee S, et al. Return on investment of the electronic vaccine intelligence network in India. *Hum Vaccin Immunother* 2022; **18**(1): 2009289.
58. Gurnani V, Singh P, Haldar P, et al. Programmatic assessment of electronic Vaccine Intelligence Network (eVIN). *PLoS One* 2020; **15**(11): e0241369.
59. Vijayalaxmi V Mogasale VM, Arindam Ray. Adverse Event Following Immunization-prevention, monitoring and reporting in India. *Indian Journal of Practical Pediatrics* 2021; **23**(1): 39-47.
60. Joshi J, Das MK, Polpakara D, Aneja S, Agarwal M, Arora NK. Vaccine Safety and Surveillance for Adverse Events Following Immunization (AEFI) in India. *Indian J Pediatr* 2018; **85**(2): 139-48.
61. MoHFW. Integrated Disease Surveillance Project. National Centre for Disease Control, Directorate General of Health Services, Ministry of Health & Family Welfare , Government of India2023. https://idsp.mohfw.gov.in/index.php (accessed.
62. coalitionagainsttyphoid.org. Typhoid Vaccines2022. https://www.coalitionagainsttyphoid.org/the-issues/typhoid-vaccines/ (accessed.
63. UNICEF. Immunization coverage by antigen (country, regional, and global trends). July 2022.

Annex 3: Survey participants by key stakeholders' groups and expertise. Expertise was self-reported by stakeholders and included multiple fields of expertise per stakeholder.

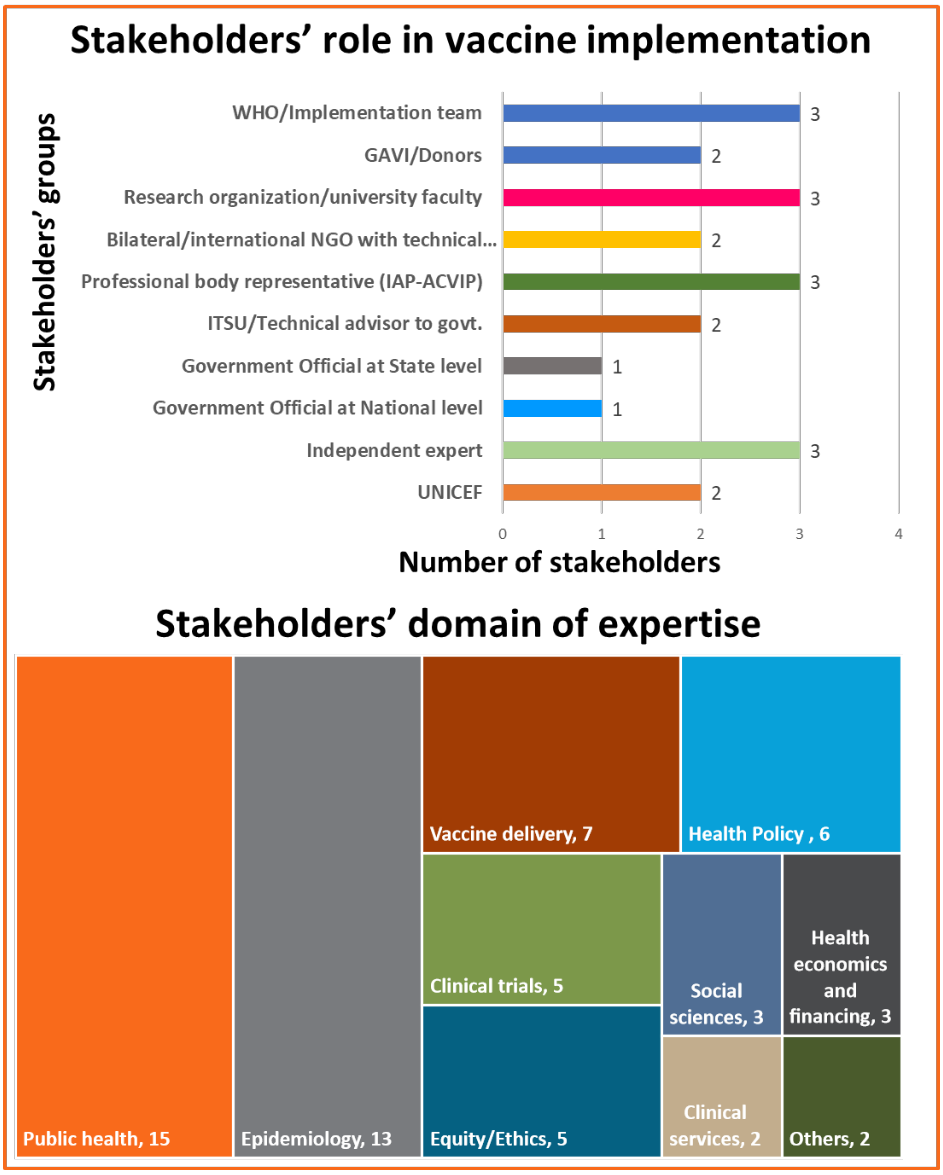

Annex 4: Stakeholder sampling and survey

Snowball sampling: First, we listed stakeholders known to the authors ("seeds") from one or more stakeholder groups. We contacted these first-line stakeholders and conducted the survey. After the survey, we requested them to refer to other stakeholders in their professional network who were in turn followed up for the survey until all different groups of stakeholders were covered. We sampled at least one person from each of the ten stakeholder groups, but not more than four per group. We ensured sufficient representation of each stakeholder group without overrepresentation of any.

Survey process: The key stakeholder survey was online supported by tele/video conferencing as required. After initial contact and willingness to participate from the potential participant, we shared the survey tool in an online form (Microsoft Forms) along with a participant information sheet (PIS) that briefly described the study, the expected average completion time, risks/benefits, and approaches for sharing results. If participants needed more guidance, a tele/video conference-based discussion was conducted at an agreed time using participants' convenient mode like Zoom/ Teams /Skype /WhatsApp call. The respondents could fill out the survey either on their computer or mobile devices. We provided specific instructions to avoid technology-related errors.

We included an informed consent form at the beginning of the survey. The participants could withdraw from participation at any time during the survey. The survey was anonymous, did not collect personal information, and only solicited stakeholders' expert or positional views on TCV introduction in India. There was no audio or video recording of the interactions/survey of stakeholders.

Annex 7: Stakeholders' responses to open-ended policy questions on optimal age of TCV introduction, state-wise roll-out of TCV, and school-based vaccination

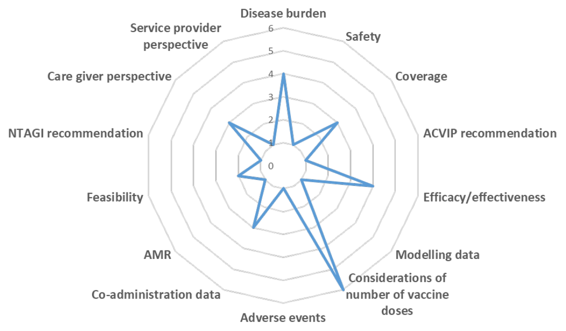

Optimal age of the TCV introduction

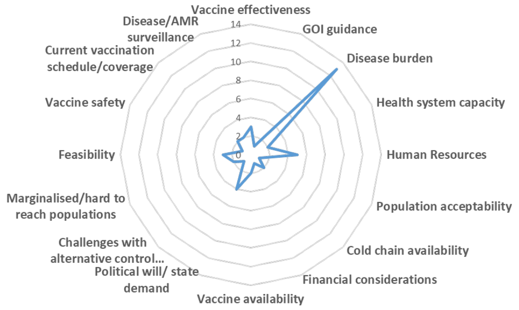

Considerations for State-wise roll-out

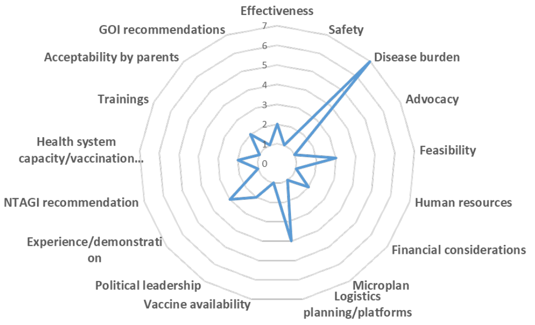

Considerations for school-based vaccination

Annex 8: Research priorities for TCV decision-making in India represented by colour chart of priority quintiles

EtR criteria	Criteria description	Evidence priorities quintile (Q) colour codes from highest (Q1) to lowest (Q5): Red=Q1, Yellow = Q2, Orange = Q3, Green= Q4, White= Q5				
The problem	Is typhoid fever a public health priority in India?	Typhoid fever incidence	Regional and international considerations	AMR tracking	Typhoid fever mortality	Severity of typhoid fever
		Alternative typhoid control measures	Socio-economic impact including treatment costs			
Benefits and harms of the intervention	Are the desirable anticipated effects large? Are the undesirable anticipated effects small?	Efficacy of vaccine	Vaccine safety	Vaccine field effectiveness	Population impact of vaccination	Duration of vaccine protection
		Co-administration safety and immunogenicity of TCV with other UIP vaccines				
Values and preferences of the target population	Are the desirable effects large relative to undesirable effects from the target population perspective?	Disease (typhoid) perception	Perception of TCV	Ethical and cultural acceptability	Demand and willingness to pay for vaccines	Vaccine schedule preferences
		Vaccine hesitancy				
Acceptability to stakeholders	Is TCV acceptable to key stakeholders (Ministry of Health, Immunisation Managers, Other national & International partners)?	Vaccine recommendation by WHO (position paper)	Gavi and donor acceptance (Financing)	Indian NTAGI acceptance (recommendation)	Professional body (IAP-ACVIP) recommendation	Immunisation managers' acceptance
		Acceptance by private practitioners	Public acceptability of vaccine			
Resource use	Are the resources required small? Cost-effectiveness (CEA) analysis.	Cost-effectiveness analysis of routine vaccination strategies	Budget required for the vaccination (budget impact analysis)	Sustainable domestic funding for vaccination	Fiscal space analysis	External funding for vaccination
Equity	What would be the impact on health inequities?	Enhanced health benefits from TCV to poor and vulnerable	Enhanced vaccine access to the poor	Financial risk protection (Decreased catastrophic expenses)	Cost-effectiveness in poor and vulnerable	
Feasibility	Is the intervention feasible to implement?	Human resources availability	Vaccine storage capacity	Robust AEFI system	Typhoid fever surveillance system	Vaccine characteristics
		Sustainable vaccine availability	Vaccination coverage under UIP	Operational feasibility of TCV co-administration with other UIP vaccines	Robust Health Management Information System	Feasibility through demonstration projects in India

AEFI= Adverse Events Following Immunisation, AMR= Antimicrobial resistance, Gavi= Gavi, the vaccine alliance, IAP-ACVIP= Indian Academy of Paediatrics-Advisory Committee on Vaccines & Immunisation Practices, NTAGI= National Technical Advisory Group on Immunisation, TCV=typhoid conjugate vaccine, UIP= Universal Immunisation Programme, WHO= World Health Organisation.

Annex 9. Evidence gaps assessed from literature versus evidence importance from the stakeholder's perspective

Evidence gap score				Evidence importance score
0 to ≤ 1	1 to ≤2	2 to ≤3	3 to ≤4	
• Severity of typhoid fever<br>• Typhoid fever incidence<br>• Efficacy of vaccine<br>• Vaccine safety<br>• Regional/International considerations<br>• Vaccine field effectiveness<br>• Cost-effectiveness of the vaccine in UIP<br>• Alternative typhoid control measures<br>• NTAGI acceptance (recommendation)<br>• Vaccine recommendation by WHO	•	• Socio-economic impact of typhoid fever<br>• Antimicrobial resistance tracking<br>• Typhoid fever mortality	• Disease perception among the target population<br>• Budget impact analysis	3 to ≤4
• Population impact of vaccination implementation<br>• Enhanced vaccine access to the poor (equity)<br>• Vaccine storage capacity<br>• Vaccine characteristics	• Enhanced health benefits from vaccination	• Cost-effectiveness in poor (equity)<br>• Duration of vaccine protection<br>• Co-administration safety and immunogenicity of vaccine<br>• Sustainable domestic funding for vaccination	• Fiscal space analysis<br>• Sustainable vaccine availability<br>• Perception of the TCV among the target population<br>• Immunisation managers' acceptance	2 to ≤3
• Human resources availability<br>• Professional body (IAP-ACVIP) acceptance<br>• Gavi/donor agency acceptability<br>• Vaccination coverage under UIP	• Feasibility of vaccine co-administer in UIP	• Typhoid fever surveillance system<br>• Financial risk protection<br>•	• Demand and willingness to pay for vaccines	1 to≤ 2
• Robust Adverse Events Following Immunisation System<br>• Robust Health Management Information System	• Public acceptability of vaccine<br>• External funding for vaccination<br>• Feasibility through vaccination demonstration project	• Acceptance by private medical practitioners<br>•	• Vaccine schedule preferences among the target population<br>• Vaccine hesitancy<br>• Ethical and cultural acceptability of vaccines	0 to ≤ 1
