# Supplementary material for: Research priorities to support typhoid conjugate vaccine decision-making in India: evidence assessment and stakeholder survey
Source: BMJ Public Health. 2024 Oct 3;2(2):e001089. doi: 10.1136/bmjph-2024-001089 (PMC11816874; doi:10.1136/bmjph-2024-001089)
Supplement: online supplemental file 2 [file bmjph-2-2-s002.pdf]

# Stakeholder Survey to Define Evidence Priorities for Typhoid Conjugate Vaccine Use in India

As a part of a PhD research (London School of Hygiene and Tropical Medicine, UK and Nagasaki University, Japan: LSHTM-NU) in collaboration with Indian Institute of Public Health, Gandhinagar (IIPHG), India, we are conducting an online survey to understand what evidences are important from a stakeholder perspective while considering Typhoid Conjugate Vaccine (TCV) introduction in routine immunisation program in India and to develop further implementation strategies. Understanding evidence priorities help us to identify evidence-gap for future research in TCV implementation in India. A larger goal of this survey is establishing an evidence-synthesis framework to support new vaccines Introduction in India. As you are the major stakeholder in vaccine implementation, we would like your opinion on the evidence priorities.

* Required

## Consent

Please read the participant information sheet sent through email before consenting. You can contact Dr. Vijayalaxmi Mogasale @ Vijayalaxmi.Mogasale@lshtm.ac.uk OR WhatsApp at +91 9448926855 for any additional information.

1. I confirm that I have read and understood the participant information sheet for "Stakeholder Survey To Define Evidence Priorities For Typhoid Conjugate Vaccine Use In India". I have had the opportunity to consider the information, ask questions, and have these answered satisfactorily.

   I understand that my consent is voluntary and that I am free to withdraw this consent at any time without giving any reason and without my rights being affected.

   I understand that expert opinion data obtained from me may be shared via a public data repository or shared directly with other researchers, and that I will not be identifiable from this personal information.

   I agree to taking part in the "Stakeholder Survey To Define Evidence Priorities For Typhoid Conjugate Vaccine Use In India". *

   ○ Yes

   ○ No

2. Please enter your email as a signature to confirm your consent *

## Role and expertise information

3. What is your role in vaccination implementation in India? (Please select all that are applicable) *

- ☐ Government Official at National level
- ☐ Government Official at State level
- ☐ Technical Advisor to Government
- ☐ Independent expert
- ☐ Bilateral, UN or international NGO with technical support role
- ☐ Professional body representative (e.g., IAP, IMA)
- ☐ Research organization/university faculty
- ☐ Donor agency staff
- ☐ Vaccination implementation team member
- ☐ Others

4. What is your area of expertise?  (Please select all that are applicable) *

- [ ] Epidemiology
- [ ] Public health
- [ ] Health Policy
- [ ] Clinical trials
- [ ] Clinical services
- [ ] Social sciences
- [ ] Health economics and financing
- [ ] Vaccine delivery
- [ ] Equity / ethics
- [ ] Others

## Evidence to Recommendation (EtR) framework

This survey uses criteria from the Evidence to Recommendation (EtR) framework developed by World Health Organization (WHO) to recommend new vaccine introduction at the country level. The survey has 8 main questions to rank the evidence listed as sub-questions. The first question lists criteria defined in the WHO EtR framework. The following 7 questions address each EtR criteria under the TCV introduction context in India. The survey also has open-ended questions that allow you to provide your suggestions.

**Instructions:** Please **click and drag** or **click and use the arrow** to move sub-questions up or down to rank them in order of importance in vaccination introduction decisions so that the most important ones are on the top. The ranking should represent the importance of the evidence from your perspective, irrespective of the availability of evidence.

**Note:** Using a device with a large screen, such as a laptop or tab, may be easiest for the survey. You may also use the mobile phone, but holding the mobile horizontally for ranking questions can help in easy navigation.

5. The criteria under WHO Evidence to Recommendation (EtR) framework for new vaccine introduction are listed randomly below. Based on your view, rank them by importance from the highest to the lowest in the Indian context. *

- **Resource use:** Is vaccination good value for money/cost-effective? Is the budget required for vaccination affordable and sustainable?
- **Disease burden**: Is the disease a public health priority in India?
- **Values and preferences of the target population:** Do the values and preferences of parents and caregivers support vaccine use? Is there a demand for the vaccine?
- **Feasibility:** Is introducing a new vaccine within a routine immunisation program feasible, accounting for challenges and opportunities?
- **Acceptability to stakeholders:** Are vaccination options acceptable to the Ministry of Health and program managers?
- **Equity:** How would health inequities impacted by the vaccine introduction in India? Will it decrease health inequity?
- **Safety and efficacy/effectiveness**: Is the vaccine safe and effective? Are the benefits larger than the harms?

6. **Optional response box:** Please let us know if you have additional suggestions on the Evidence to Recommendation (EtR) framework criteria considering the Indian context.

## Criteria: Public health priority (Typhoid disease burden)

7. If you have accounted **typhoid disease burden** as one of the criteria for TCV introduction decision in India, which of the following evidence sub-question do you consider most important under this category? Rank below listed evidence sub-questions from highest importance to lowest. *

- **AMR:** As antimicrobial resistance (AMR) in *S*. Typhi limits treatment options and increases its potential for spread, is tracking AMR a priority in India?
- **Socio-economic impact:** Are the health care costs to the government and households and loss of workdays due to typhoid high in India?
- **Typhoid incidence:** Is the typhoid incidence in India high enough to consider it a priority disease?
- **Disease severity:** Are typhoid complications and hospitalisation rates high in the Indian population?
- **Mortality:** Is typhoid-related death high in the Indian population?
- **Alternative typhoid control measures:** Are health education, improving hygiene, and food handling practices practical, effective and affordable in India?
- **Regional/International considerations:** Is typhoid burden high in neighbouring and other low and middle income countries with a potential to spread make it a priority in India?

8. **Optional response box:** Please recommend if you think of any additional elements to be considered under typhoid disease burden criteria. Also, please let us know if you prefer to rank any of the above-listed sub-questions at the same level or if any of them do not apply to the Indian context.

## Criteria: Benefits and harms of TCV intervention

9. If you have accounted **benefits and harms of TCV intervention** as one of the criteria for TCV introduction decision in India, which of the following evidence sub-question do you consider most important under this category? Rank below listed evidence sub-questions from highest importance to lowest. *

- **Efficacy:** Does TCV show good efficacy based on clinical trials?
- **Safety:** Is TCV shown to be safe based on clinical trials?
- **Field effectiveness:** Does the demonstration project show TCV is safe and effective in field situations?
- **Co-administration safety and immunogenicity:** Is TCV co-administration with other EPI vaccines (e.g., MR, IPV, JE, DPT) safe and immunogenic?
- **Duration of protection:** Does TCV offer long-term protection?
- **Population impact:** Does TCV introduction have a large protective effect (including herd protection) at the population level to control typhoid?

10. **Optional response box:** Please recommend if you think of any additional elements to be considered under benefits and harms of TCV intervention criteria. Also, please let us know if you prefer to rank any of the above-listed sub-questions at the same level or if any of them do not apply to the Indian context.

## Criteria: Values and preferences of the target population

The target population is defined as parents and caregivers of children.

11. If you have accounted **values and preferences of the target population** as one of the criteria for TCV introduction decision in India, which of the following evidence sub-question do you consider most important under this category? Rank below listed evidence sub-questions from highest importance to lowest. *

- **Disease perception:** How does the target population feel the risk of getting typhoid fever among their children to consider the vaccination?
- **Perception of TCV:** How does the target population value or perceive the desirable and undesirable effects of the TCV introduction?
- **Ethical and cultural acceptability:** Does the target population perceive TCV as an acceptable intervention ethically and culturally?
- **Demand and willingness to pay for vaccines:** How much money are parents willing to pay for TCV, and when introduced in the routine immunisation program, will there be demand?
- **Schedule preferences:** Considering multiple injections under the EPI schedule, do parents prefer TCV at 9-12 months or 16-24 months?
- **Vaccine hesitancy:** Does target population perception indicate a potential risk for TCV hesitancy?

12. **Optional response box:** Please recommend if you think of any additional elements to be considered under values and preferences of the target population criteria. Also, please let us know if you prefer to rank any of the above-listed sub-questions at the same level or if any of them do not apply to the Indian context.

## Criteria: Acceptability of TCV intervention to stakeholders

The acceptability of vaccine introduction for ministry of health and program managers is a multidimensional consideration (e.g. ethical, programmatic, financial etc.). The acceptance can also stem from different Indian and global stakeholders.

13. If you have accounted **acceptability to stakeholders** as one of the criteria for TCV introduction decision in India, which of the following evidence sub-question do you consider most important under this category? Rank below listed evidence sub-questions from highest importance to lowest. *

- **WHO:** Is TCV recommended by WHO?
- **Gavi/donor agency:** Has Gavi included TCV in its vaccine portfolio? Are donors ready to support TCV introduction in India?
- **NTAGI:** Is the National Technical Group on Immunisation in India (NTAGI) recommended TCV introduction in India?
- **Professional body:** Is professional body like Indian Academy Paediatrics (IAP) recommended TCV use?
- **Immunisation managers:** Do immunisation managers accept TCV as an additional vaccine in the routine immunisation schedule?
- **Private medical practitioners:** Is TCV valued by private medical practitioners and accepted in the private market?
- **Public acceptability:** Does the demonstration project show the programmatic and public acceptability of TCV?

14. **Optional response box:** Please recommend if you think of any additional elements to be considered under acceptability to stakeholders criteria. Also, please let us know if you prefer to rank any of the above-listed sub-questions at the same level or if any of them do not apply to the Indian context.

## Criteria: Resource use

Resource use considerations include if intervention is a reasonable and efficient allocation of resources, good value for money, and sustainable.

15. If you have accounted **resource use** as one of the criteria for TCV introduction decision in India, which of the following evidence sub-question do you consider most important under this category? Rank below listed evidence sub-questions from highest importance to lowest. *

- **Cost-effectiveness:** Is TCV introduction in routine immunisation in India a good value for money?
- **Budget requirement:** How much money is needed for the routine introduction of TCV in India? Is it large?
- **Sustainable funding:** Is there a domestic (government) financial commitment that can sustainably support TCV in the future?
- **Fiscal space analysis:** Does the health budget in India has space to accommodate the budget of introducing TCV? In other words, is there enough money for the TCV introduction?
- **External funding:** Are Gavi or other donors willing to fund TCV implementation in India?

16. **Optional response box:** Please recommend if you think of any additional elements to be considered under resource use criteria. Also, please let us know if you prefer to rank any of the above-listed sub-questions at the same level or if any of them do not apply to the Indian context.

## Criteria: Health equity

Health equity means accounting for the benefits to poor and vulnerable people (for example, people with low income, slum dwellings, marginalised population groups).

17. If you have accounted **health equity** as one of the criteria for TCV introduction decision in India, which of the following evidence sub-question do you consider most important under this category? Rank below listed evidence sub-questions from highest importance to lowest. *

**Increased health benefits:** Does TCV introduction offer improve health benefits to the poor, vulnerable, or people living in urban slums?

**Financial risk protection:** Does TCV introduction decreases catastrophic health care expenses and protect from financial risks in the population?

**Enhanced vaccine access:** Does the routine introduction of TCV improve current TCV coverage in poor, vulnerable, or people living in urban slums, as now it is limited to the private market?

**Cost-effective in poor:** Is TCV introduction a good value for money for the poor, vulnerable, or people living in urban slums?

18. **Optional response box:** Please recommend if you think of any additional elements to be considered under health equity criteria. Also, please let us know if you prefer to rank any of the above-listed sub-questions at the same level or if any of them do not apply to the Indian context.

## Criteria: Feasibility

19. If you have accounted **feasibility of vaccination** as one of the criteria for TCV introduction decision in India, which of the following evidence sub-question do you consider most important under this category? Rank below listed evidence sub-questions from highest importance to lowest. *

- **Human resources:** Are the available human resources sufficient?
- **Vaccine storage capacity:** Is available cold chain capacity sufficient?
- **AEFI monitoring:** Is the Adverse Events Following Immunisation system robust enough to track post-introduction TCV AEFI?
- **HMIS:** Is Health Management Information System robust enough to track coverage and utilisation of TCV?
- **Infectious disease surveillance system:** Is the surveillance system robust enough to monitor post-TCV introduction typhoid cases?
- **Vaccine characteristics:** Does TCV presentation and route of administration make it feasible to deliver the vaccine efficiently by service providers?
- **Vaccine availability:** Is there sustainable supply of TCV in India?
- **Vaccination coverage**: Is current EPI vaccine coverage high enough to accommodate the additional load of TCV introduction?
- **Co-administration:** Is it feasible to co-administer TCV with other EPI vaccines (e.g., DPT, MR, IPV, JE)?
- **Demonstration project:** Is the feasibility of TCV implementation established in field settings?

20. **Optional response box:** Please recommend if you think of any additional elements to be considered under feasibility criteria. Also, please let us know if you prefer to rank any of the above-listed sub-questions at the same level or if any of them do not apply to the Indian context.

## Other considerations for TCV implementation in India

21. What evidence is important (irrespective of availability) to decide the **optimal age schedule** for TCV introduction under EPI? Two potential options are at 9 -12 months with the measles-containing vaccine (MCV) or at 16-24 months with a DPT booster dose. Please let us know a list of important evidence based on your view. *

22. What evidence is important (irrespective of availability) to decide the **state-wise rollout** of TCV in India? Please let us know a list of important evidence based on your view. *

23. What evidence is important (irrespective of availability) to decide on the **school-based vaccination** to deliver an additional dose of TCV in India? Please let us know a list of important evidence based on your view. *

## Confirmation

Thank you very much for completing the survey. We appreciate your opinion and value the time spent on this survey.

24. I confirm submission of my response

- ◯ Yes, I confirm
- ◯ No, I withdraw from the survey

This content is neither created nor endorsed by Microsoft. The data you submit will be sent to the form owner.

Microsoft Forms
